# *In Vitro* Gene Silencing of the Fish Microsporidian *Heterosporis saurida* by RNA Interference

**DOI:** 10.1089/nat.2016.0613

**Published:** 2016-08-01

**Authors:** Mona Saleh, Gokhlesh Kumar, Abdel-Azeem Abdel-Baki, Mohamed A. Dkhil, Mansour El-Matbouli, Saleh Al-Quraishy

**Affiliations:** ^1^ Clinical Division of Fish Medicine, University of Veterinary Medicine, Vienna, Austria.; ^2^ Zoology Department, College of Science, King Saud University, Riyadh, Saudi Arabia.; ^3^ Zoology Department, Faculty of Science, Beni-Suef University, Beni-Suef, Egypt.; ^4^ Department of Zoology and Entomology, Faculty of Science, Helwan University, Cairo, Egypt.

## Abstract

*Heterosporis saurida*, a microsporidian parasite of lizardfish, *Saurida undosquamis*, causes severe economic losses in marine aquaculture. Among the novel approaches being explored for treatment of parasitic infections in aquaculture is small interfering RNA molecules. The aim of the present study was to investigate the efficiency of using siRNA to knock down expression of specific genes of *H. saurida in vitro*. For this purpose, siRNAs specific for ATP/ADP antiporter 1 and methionine aminopeptidase II genes were designed and tested using a previously developed *in vitro* cultivation model. Silencing of *H. saurida* target genes was assessed and the efficacy of using siRNA for inhibition of gene expression was measured by quantitative real-time polymerase chain reaction (PCR). Silencing of ATP/ADP antiporter 1 or methionine aminopeptidase II by siRNA reduced *H. saurida* infection levels in EK-1 cells 40% and 60%, respectively, as measured by qRT-PCR and spore counts. Combined siRNA treatment of both ATP/ADP antiporter 1 and methionine aminopeptidase II siRNAs was more effective against *H. saurida* infection as seen by the 16S rRNA level and spore counts. Our study concluded that siRNA could be used to advance development of novel approaches to inhibit *H. saurida* and provide an alternative approach to combat microsporidia.

## Introduction

Worldwide, diseases of aquatic animals impose considerable constraints to the expansion and management of aquaculture, thus attempts to control diseases have become a main concern in many fish-producing areas. Microsporidia are obligate, protozoan, intracellular, parasites that infect a broad range of animals, including fish, and are increasingly recognized as economically and medically important parasites [1]. Microsporidian infections by members of genus *Heterosporis* may lead to major pathogenic effects to their hosts. Unlike many other microsporidia, *Heterosporis* spp. do not produce a xenoma, but infect tissues diffusely, and may become bordered by host connective tissue [2].

*Heterosporis saurida* isolated from lizardfish, *Saurida undosquamis* in the Arabian Gulf, causes significant pathogenic effects on the host. Fish muscle cells are destroyed and replaced by connective tissue, resulting in a thin or concave exterior and leads to a freezer-burn appearance, which makes the fish unfit for human consumption and leads to negative economic consequences on trade in this fish species [3]. However, the molecular basis of microsporidian pathogenicity and virulence is largely unexplored, due, in part, to the scarceness of suitable systems to support studies of host–pathogen interactions and allow genetic manipulation [1,4].

Methionine aminopeptidase II is an ubiquitously expressed enzyme that plays a critical role in cell development and differentiation. It is involved in the regulation of protein synthesis and posttranslational processing. Fumagillin, a methionine aminopeptidase II inhibitor, is generally useful against microsporidiosis, but it is toxic when administered systemically to mammals [5]. Toxic effects have been also reported in fish exposed to higher doses of fumagillin. Direct mortality and histological examination revealed extensive toxic alteration in the liver and posterior kidney [6]. Furthermore, necrosis in the interstitial tissue, degeneration of the epithelial cells of the tubules, and a reduction in melanomacrophage center numbers were reported [7].

To avoid development of antibiotic resistance and reduce toxicity associated with drug application, therapeutic alternatives for microsporidiosis should be explored. A potential molecular target in intracellular parasites such as *Rickettsia* spp. and the microsporidian *Nosema ceranae* is a nucleotide transporter that imports ATP from host cells. It has been reported that double-stranded RNA homologous to specific *N. ceranae* ADP/ATP transporter genes can specifically and differentially silence transcripts that encode these proteins. This inhibition was found to affect *Nosema* levels and host physiology [8].

Overall, exploration of host–parasite interactions and the associated molecular events of fish parasites have been hampered due to lack of suitable *in vitro* systems. Recently, an *in vitro* cultivation model using an eel kidney cell line (EK-1) that is susceptible to infection by *H. saurida* was developed [9]. This *in vitro* system has been used successfully as a model for screening and development of drugs and enables us to conduct RNA interference (RNAi) experiments [10]. RNAi is a natural mechanism for posttranscriptional gene silencing triggered by small interfering RNA (siRNA). This technique is not only used as a potential tool in investigating the functional role of genes of interest, but also to repress disease and growth of many pathogens that cause severe ecological and economical losses [11].

Our group has successfully applied the siRNA approach to prove the efficacy of such strategy to manipulate and combat the fish pathogens; cyprinid herpesvirus-3, spring viremia of carp virus, and *Myxobolus cerebralis* [12–14]. Herein, we explored the efficacy of the siRNA approach to knock down ADP/ATP antiporter 1 gene of *H. saurida*. Additionally, we investigated whether siRNA specific for *H. saurida* methionine aminopeptidase II could manipulate the parasite to cause specific gene silencing and reduce spore counts. Accordingly, we designed siRNAs to silence *H. saurida* ADP/ATP antiporter 1 and methionine aminopeptidase II genes and tested them *in vitro* utilizing an eel kidney cell line (EK-1).

The knockdown efficiency of the siRNAs was evaluated by quantitative real-time polymerase chain reaction (qRT-PCR). The inhibition of *H. saurida* was measured by quantifying the expression of 16S rRNA using qRT-PCR and by spore counts in EK-1 cell culture. To the best of our knowledge, this is the first gene silencing investigation of a fish microsporidian parasite. Our results may lead to the development of novel approaches to protect fish from microsporidia.

## Materials and Methods

### Preparation of spores

**FIG. 1.**
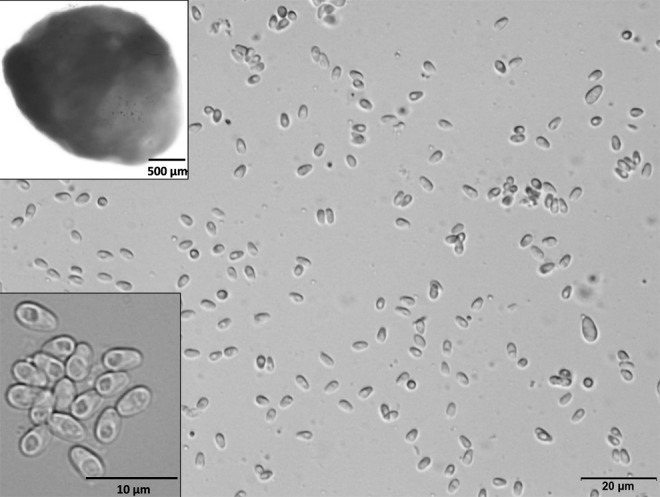
Free fresh spores of *Heterosporis saurida*, which were released from a cyst (*upper left*) in the muscle of naturally infected lizardfish, *Saurida undosquamis*. More highly magnified spores are shown *lower left*.

*H. saurida* spores (Fig. 1) were prepared as previously described [10]. Briefly, to inactivate bacteria, spores were treated with 10,000 U per mL penicillin and 10,000 μg per mL streptomycin in L-15 medium for 2 h at 37°C. Spore pellets were washed twice with serum-free L-15 (Leibovitz) medium with l-glutamine (Sigma-Aldrich), and then resuspended in serum-free L-15 medium. Spores were counted through a hemocytometer to achieve a concentration of 1 × 10^7^ spores per mL.

### Growth of eel kidney epithelial cells (EK-1)

Eel kidney epithelial cells (EK-1) were grown at 26°C and maintained in L-15 medium with l-glutamine, and 10% fetal bovine serum (FBS; Invitrogen) [15]. Penicillin and streptomycin (Sigma-Aldrich) were supplemented at 100 IU per mL and 100 μg per mL, respectively. For preparation of *H. saurida*-infected cells, EK-1 cells were seeded into 24-well plates at 1 × 10^5^ cells per well. Confluent cultures were challenged with *H. saurida* spores at a concentration of 10^7^ spores per mL to achieve a final ratio of 3:1 spores/cell [10].

### siRNA design and sequences

Gene sequences homologous to ATP/ADP transporters and methionine aminopeptidase II of *H. saurida* were used (unpublished data). Small interfering double-stranded RNAs to target *H. saurida* ADP/ATP antiporter 1 and methionine aminopeptidase II genes were designed using Silencer Select Designer (www.thermofisher.com/at/en/home/life-science/rnai/synthetic-rnai-analysis/ambion-silencer-select-sirnas.html). Different siRNAs to target ADP/ATP antiporter 1 and methionine aminopeptidase II genes were selected. The highest-ranking sense and antisense siRNA duplexes that showed the best activity and specificity were synthesized by Ambion (Invitrogen) at 5 nmoles concentration as lyophilized powder. Stock solutions (100 μM) were prepared and used at 2 μM final concentration, or stored at −20°C until use.

siRNAs were used singly or equally mixed in transfection experiments. The 5′–3′ sequences of the sense and antisense siRNA strands were (GCGUCAAACUGAACAAAGATT), (UCUUUGUUCAGUUUGACGCTT) and (CCGAUGUGCUGAAAAUUGATT), (UCAAUUUUCAGCACAUCGGTT) for ADP/ATP antiporter 1 and methionine aminopeptidase II genes, respectively.

### Optimization of transfection efficiency

According to the manufacturer's instructions, 1 μm, 1.5 and 2 μM of the silencer FAM-labeled negative control No. 1 siRNA (#AM 4620; Ambion) were mixed with equal volumes of Lipofectamine RNAiMAX (Invitrogen). The silencer FAM-labeled negative control siRNA was resuspended in Opti-MEM reduced-serum medium aliquots (Invitrogen) and incubated with Lipofectamine RNAiMAX reagent at room temperature for 30 min. EK-1 cells were seeded into 24-well plates at 1 × 10^5^ cells per well in serum-free L-15 medium.

Cultures were allowed to incubate with the silencer FAM-labeled negative control with siRNA-Lipofectamine RNAiMAX complexes for 4 h at 26°C, media were replaced with L-15 medium containing 10% FBS, and then cells were observed daily using fluorescence microscopy. Confluent cell cultures were challenged with *H. saurida* spores at a final ratio of 3:1 spores/cell, incubated with siRNA-Lipofectamine RNAiMAX for 4 h at 26°C, and further incubated for 24 h at 26°C before any nonadherent spores were rinsed off. Media were replaced with fresh media containing 10% FBS and cultures were monitored daily using fluorescence microscopy. Six wells were evaluated for each tested concentration and control cells at the different time points in triplicates.

### Assessment of transfection efficiency

**FIG. 2.**
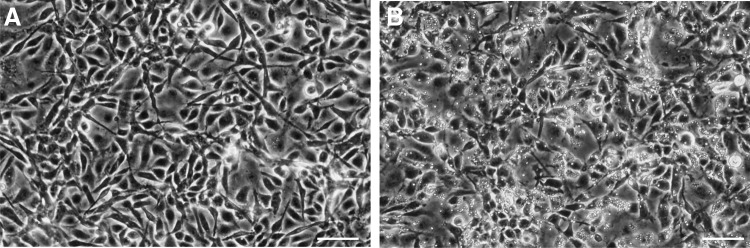
Eel kidney epithelial (EK-1) cells, both uninfected and infected with *H. saurida*. **(A)** Uninfected EK-1 cells, scale bar 20 μm, **(B)** EK-1 cells infected with *H. saurida*, scale bar 50 μm.

EK-1 cells were subcultured and seeded into 24-well plates and *H. saurida* spores were added at a concentration of 10^7^ spores/mL to achieve a final ratio of 3:1 spores/cell. After 24 h, noninternalized or nonadherent spores were washed off, and fresh medium was added and incubated at 26°C (Fig. 2). According to the manufacturer's instructions, using the silencer FAM-labeled negative control, the efficiency of the siRNA delivery was assessed in EK-1 cell cultures at 1, 1.5, and 2 μM concentrations at 24, 48, and 72 h postinfection.

Accordingly, EK-1 cells infected with *H. saurida* spores incubated in serum-free L-15 medium were transfected with a solution containing 2 μM FAM-labeled negative control siRNA for 4 h at 26°C. After washing off the uninternalized spores, fresh medium containing 10% FBS was added. The siRNA uptake into *H. saurida* in EK-1 was observed after incubation with the FAM-labeled negative control siRNA at 2 μM concentrations at 26°C. Six wells were evaluated at each time point in triplicates.

### Gene knockdown

EK-1 cells were transfected with 2 μM of siRNA and 2 μL of Lipofectamine RNAiMAX per well as described above. Briefly, siRNA single (2 μM) or combined (1 or 2 μM each siRNA) siRNA was resuspended in Opti-MEM reduced-serum medium aliquots and incubated with Lipofectamine RNAiMAX reagent at room temperature for 30 min. Each siRNA treatment was applied to individual wells. Cells were then seeded into the 24-well plates at 1 × 10^5^ cells per well in serum-free L-15 medium.

Cultures were allowed to incubate with siRNA–Lipofectamine RNAiMAX complexes for 4 h at 26°C. Confluent cultures were infected with *H. saurida* spores and further incubated for 24 h at 26°C. Unattached spores were washed off and fresh L-15 media supplemented with 10% FBS were added and checked daily with fluorescence microscopy. Cells were collected from six wells in triplicates for RNA extraction at 24 h, 72 h, and 7 days postinfection for qRT-PCR measurement of siRNA gene knockdown.

### RNA extraction and qRT-PCR

Total RNA was isolated from siRNA-treated and nontreated infected cells using the RNeasy Mini Kit (Qiagen) according to the manufacturer's instructions. An on-column DNase digestion step was included and RNA samples were quantified using a NanoDrop 2000 spectrophotometer (Thermo Scientific). Using an iScript cDNA Synthesis Kit (Bio-Rad), cDNA was synthesized with 1 μg total RNA as per the user's manual. The efficiency of gene knockdown was evaluated by quantitative PCR (qPCR). Primers were designed (Table 1) and each primer set was tested to determine the optimal annealing temperature and primer concentration. PCR amplifications were completed using specifically designed primers. Real-time qPCR was performed using a Bio-Rad iCycler according to the manufacturer's instructions, in a final volume of 20 μL:4 μL of 1:2-fold diluted cDNA, 10 pmol of each primer, 2× SsoAdvanced Universal SYBR Green Supermix (Bio-Rad), and DEPC-treated sterile distilled water.

**Table 1.** Primers Used for Quantitative Real-Time Polymerase Chain Reaction

*Primer name*	*Gene target*	*Sequence (5′–3′)*	*Annealing temperature (°C)*	*Amplicon size (bp)*
ATP F	ATP/ADP	AGCCTTCATTTTCCGAAGGA	55	186
ATP R	antiporter 1	GCGCAGACATGAAAAGGACA		
Methionine F	Methionine aminopeptidase	GCGCCAGGAATGTCTCTTAG	55	127
Methionine R	II	GTGCTGCACAGTCGTTCAAA		
16S F	16S rRNA	GCACGAGTGTTGTACACGA	55	104
16S R		GACAGCAGGGGTCTCACTAC		

Each qRT-PCR was performed in triplicate. After 5 min of cDNA denaturation at 95°C, 38 cycles were performed at 95°C for 30 s, 55°C for 30 s, and 72°C for 30 s. Standard curves were constructed for the target genes to measure the quantity of each target genes in tested samples. Expression levels were assessed at each time point utilizing statistical analyses. Differences between groups were analyzed using t tests for samples with Bonferroni α-correction, and differences between time points were analyzed using paired *t*-tests with SPSS software version-20. For all statistical tests, a *p* ≤ 0.05 was considered as significant.

### H. saurida spore counts

Counting of *H. saurida* spores in infected EK-1 was performed as previously described [8]. Briefly, spores were added to 1 mL volumes of medium at a concentration of 10^7^ spores/mL to achieve a final ratio of 3:1 spores/cell. After 24 h, noninternalized or nonadherent spores were washed off, and fresh medium was added and incubated at 26°C for 7 days.

Cell monolayers were examined under an inverted microscope and 10 cells in 10 fields were examined per well (six wells of each siRNA-treated and nontreated infected cells). Each assay was repeated three times. siRNA applications were assayed in triplicate, and the percent inhibition of *H. saurida* replication was calculated: percent inhibition = 100 − [(mean number of parasites counted in treated cultures/mean number of parasites counted in nontreated infected cultures) ×100]. The differences between spore counts of siRNA-treated and nontreated infected wells were analyzed using *t*-tests with Bonferroni α-correction.

## Results

### Assessment of transfection efficiency

**FIG. 3.**
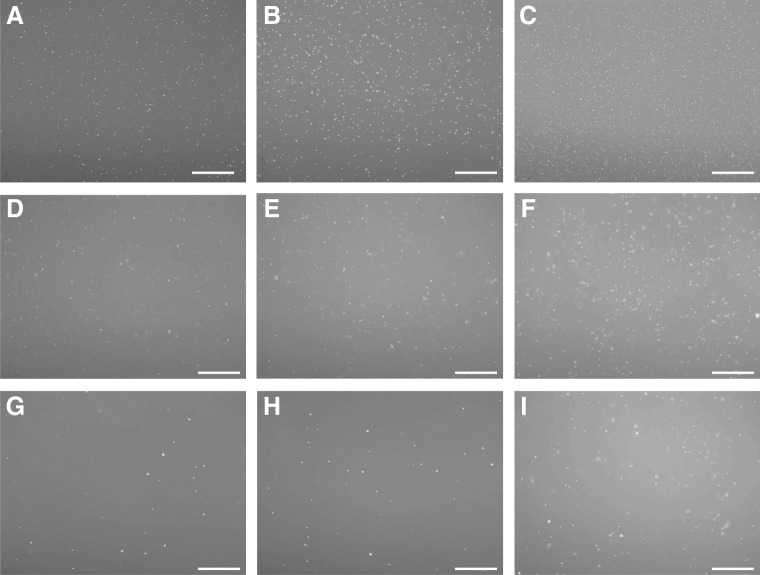
siRNA uptake into EK-1 was observed after incubating at 26°C for 24 h **(A–C)**, 48 h **(D–F),** and 72 h **(G–I)** with a FAM-labeled negative control siRNA at 1, 1.5, and 2 μM. Scale bar 50 μm.

**FIG. 4.**
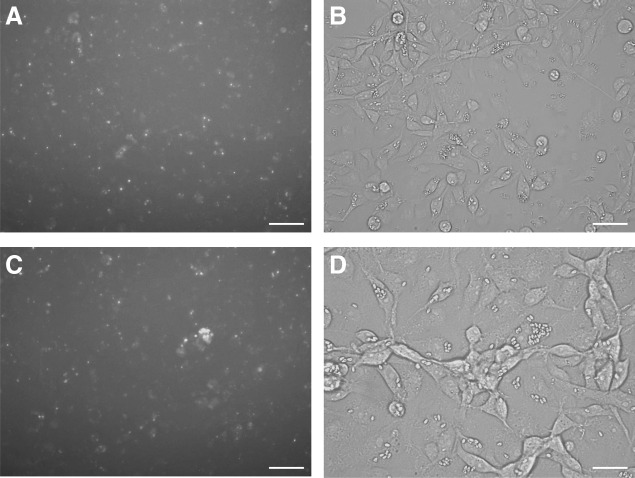
FAM-labeled negative control siRNA uptake into *H. saurida* in EK-1 cells was observed with a fluorescence inverted microscope after incubating at 26°C for 72 h, as shown in **(A, B)** and **(C, D)**, scale bar 50 μm.

Using the silencer FAM-labeled negative control, the optimal efficiency of siRNA delivery was observed in EK-1 cells at 2 μM (Fig. 3A–I). EK-1 cells infected with *H. saurida* spores incubated in serum-free L-15 medium were transfected accordingly with a solution containing 2 μM FAM-labeled negative control siRNA and the uptake into *H. saurida* in EK-1 was observed (Fig. 4).

### Validation of gene knockdown by qRT-PCR

**FIG. 5.**
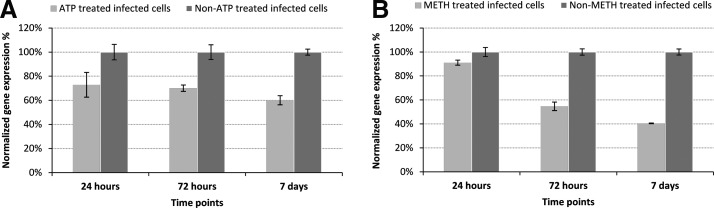
Gene expression of siRNA treated and untreated infected samples at different time points. **(A)** ATP/ADP antiporter 1 gene, **(B)** methionine aminopeptidase II gene. mRNA expression levels of ATP/ADP antiporter 1 and methionine aminopeptidase II genes were knocked down 40% and 60% at 7 days postinfection, respectively.

qPCR showed that there was a significant reduction (*p* < 0.0001 or *p* < 0.0009) of expression of the targeted genes at three different time points in three separate experiments. Expression of both siRNA ATP/ADP antiporter 1 and methionine aminopeptidase II treatments was reduced up to 40% and 60% at 7 days postinfection, respectively (Fig. 5). This confirmed that siRNA specifically targeted *H. saurida* genes in infected EK-1 cells. Quantification data are shown in Fig. 4. Methionine aminopeptidase II was more effectively inhibited than ATP/ADP antiporter 1 at 48 h and 7 days postinfection.

### Effect of siRNA treatments on H. saurida infection levels and spore counts

**FIG. 6.**
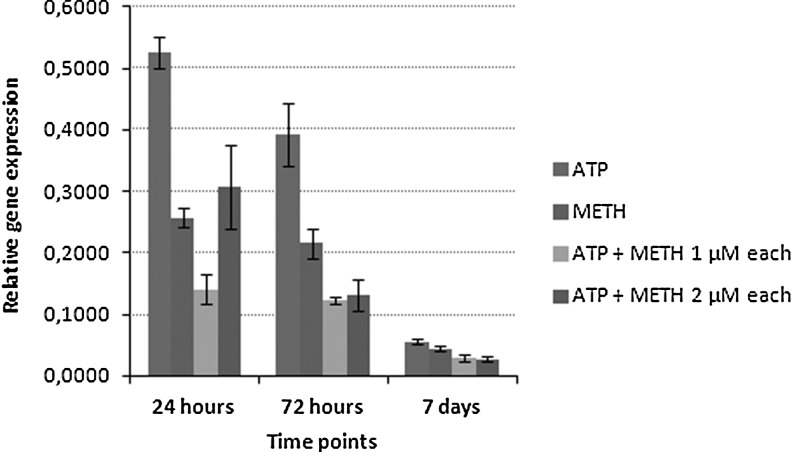
Relative gene expression of *H. saurida* 16S rRNA in response to single and combined siRNA treatments at different time points. At 7 days postinfection, *H. saurida*-infected EK-1 cells incubated with single or combined siRNAs consistently showed the lowest infection levels, as measured by transcript abundance of *H. saurida* 16S rRNA.

EK-1 cells incubated with single or combined siRNA treatments consistently showed lower infection levels than untreated cells, as measured by expression levels of *H. saurida* 16S rRNA at 24 h, 72 h, and 7 days postinfection (Fig. 6). Quantification data are in Fig. 5. Combined siRNA treatments of ATP/ADP antiporter 1 and methionine aminopeptidase II also reduced propagation of spore at each time points. Spore counts indicated that EK-1 incubated with the single or combined siRNA had lower infection levels than nontreated, infected cells (Table 2).

**Table 2.** Effects of Used siRNAs on *Heterosporis saurida* in EK-1 Cells

*siRNA treatments*	*Average numbers of H. saurida (6 wells of 10 infected EK-1 cells in 10 fields)*	*Average% inhibition 7 days postinfection*
ATP/ADP antiporter 1	62.5 ± 3.1	40.47 ± 4
Methionine aminopeptidase II	42.5 ± 2.7	59.53 ± 6
Combined siRNA (1 μM each siRNA)	39.6 ± 4.3	62.29 ± 3
Combined siRNA (2 μM each siRNA)	37.1 ± 2.3	64.67 ± 4
Untreated infected cells	105.0 ± 3.7	—

## Discussion

Worldwide, parasitic diseases of fish attract attention since they impact the health of populations and affect food fish production. Microsporidian parasites infect most animal groups, including fish, frequently with devastating consequences for the host [16–18]. *H. saurida* cause significant pathogenic effects on the host and consequently have negative economic results on the fish trade. Moreover, *H. saurida* infects fish tissues diffusely making them unsuitable for eating [3].

Microsporidia are unable to synthesize the building blocks of DNA and RNA for themselves, and must import these materials from the infected host [19]. Due to the major loss of genes affecting most metabolic pathways, transport proteins are vital for completing the microsporidian life cycle inside infected host cells [20,21]. ADP/ATP transporter genes have been reported to be significant and probably crucial component in providing the essential biochemical and metabolic requirements of microsporidian parasites [8,22]. To target the main energy highway and minimize potential off-target effects, ADP/ATP antiporter 1 of *H. saurida* has been preferred as a target for siRNA experiments [8].

Fumagillin is an irreversible methionine aminopeptidase II inhibitor that demonstrates activity against microsporidiosis [23]. The inhibitory action of this drug is due to its ability to covalently bind to a histidine residue in the active site of methionine aminopeptidase II [24,25]. Methionine aminopeptidase II are enzymes responsible for the removal of methionine from the amino-terminus of newly synthesized proteins [26]. Removal of methionine is crucial for further amino-terminal modifications and for protein stability [27].

Fumagillin was effective against the microsporidian *Heterosporis anguillarum* in eels (*Anguilla japonica*) and against *Loma salmonae* in salmon [28,29]. Recently, fumagillin was identified as a promising therapeutic agent against *H. saurida* [10]. These studies suggest that methionine aminopeptidase II is suitable molecular target of *H. saurida*. Herein, we investigated the efficacy of using small interfering RNA to knockdown the expression of methionine aminopeptidase II, as well as ADP/ATP antiporter 1 genes of *H. saurida*.

Recently, RNAi has emerged as a powerful tool to silence the expression of genes and analyze their loss-of-function phenotype, yet few studies have been performed to manipulate and study gene function of fish parasites. Functional analysis of parasite genes shows a strong potential to assist in identification and characterization of new drug and vaccine candidates against fish parasitic diseases [30–33]. siRNA approaches have been recently utilized to combat various pathogens that affect fish [11]. We have investigated the potential for siRNAs to inhibit viral replication of cyprinid herpesvirus-3 in common carp brain cells [12]. siRNAs were designed to target two genes implicated in DNA replication, thymidine kinase and DNA polymerase. The inhibition of viral replication after knockdown by siRNAs was measured using a reporter gene (ORF81).

In addition, siRNAs designed to target SVCV-N and SVCV-P transcripts inhibited spring viremia of carp replication *in vitro* in an epithelioma papulosum cyprinid cell line [13]. siRNA knockdown of phosphoprotein or nucleoprotein reduces *in vitro* viral replication, with use in tandem worked best. Furthermore, this technique has been investigated regarding its effectiveness to control whirling disease [14]. Salmonid whirling disease is caused by the myxozoan parasite, *M. Cerebralis*, and alternates between two hosts, a salmonid fish and an invertebrate oligochaete, *Tubifex tubifex*. siRNA that targeted *MyxSP-1* was found to induce *in vivo* RNAi that leads to abrogation of the *M. cerebralis* life cycle in the oligochaete host.

In the present study, we demonstrated that siRNA can inhibit propagation and development of the intracellular microsporidian parasite *H. saurida*. We used an siRNA approach to target both ATP/ADP antiporter 1 and methionine aminopeptidase II genes of the parasite *in vitro* in EK-1 cells. To optimize transfection efficiency, we used the silencer FAM-labeled negative control No. 1 siRNA (#AM 4620; Ambion), which is unrelated to *H. saurida* and *A. japonica* and does not target any gene products. Additionally, this control does not have any sequence homology to *Mus musculus*, *Rattus norvegicus*, and *Homo sapiens*, and has been screened by the manufacturer in cell cultures, and confirmed to have no significant effect on cell proliferation, viability, or morphology. We observed that siRNA delivery was optimal at 2 μM, thus this concentration was used in all subsequent experiments.

mRNA expression levels of ATP/ADP antiporter 1 and methionine aminopeptidase II genes were knocked down 40% and 60% at 7 days postinfection, respectively. *H. saurida* incubated with siRNA specific to ATP/ADP antiporter 1 and methionine aminopeptidase II had lower spore counts than nontreated infected cells. Furthermore, *H. saurida*-infected Ek-1 cells incubated with single or combined siRNAs consistently showed lower infection levels, as measured by transcript abundance of *H. saurida* 16S rRNA and spore counts. The specific silencing of ATP/ADP antiporter 1 gene appeared to level off after 7 days; as observed in a similar study with *N. ceranae* [8]. This reduction of efficacy may arise from a compensatory mechanism in *H. saurida*, as has been proposed for *N. ceranae* [8].

The specific silencing of methionine aminopeptidase II gene in single or combined siRNA applications appeared more stable. These results are novel for this microsporidian and provide a possible avenue for controlling disease in lizardfish. Use of RNAi represents a significant advance toward molecular control and characterization of new vaccine candidates against fish parasitic diseases. Future genomic and proteomic studies of *H. saurida* should help to identify additional targets for silencing (eg, virulence genes). The results we present here suggest that siRNA could be applied to explore gene function of other microsporidia beyond those affecting fish, as microsporidian parasites are implicated in a wide range of veterinary and human diseases.
